
JOURNAL INFORMATION
==============================
NLM Title Abbreviation: J Orthop Traumatol
Journal ID: J Orthop Traumatol
NLM Journal ID: 101090931

Journal of Orthopaedics and Traumatology : Official Journal of the Italian Society of Orthopaedics and Traumatology

ISSN: 1590-9921
EISSN: 1590-9999
Publisher: Springer

ARTICLE INFORMATION
==============================
PMCID: PMC4417842
DOI: 10.1007/s10195-011-0171-x
Article ID: 171
Article version: 1
Subjects: Acknowledgement to Referees

2011 Scientific Referees

Electronic publication date: 2011 Nov 29
Print publication date: 2011 Dec
Volume: 12
Issue: 4
First page: 227
Last page: 228
Copyright: © The Author(s) 2011
License: This article is distributed under the terms of the Creative Commons Attribution License which permits any use, distribution and reproduction in any medium, provided the original author(s) and source are credited.
License URL: https://creativecommons.org/licenses/by/4.0/

issue-copyright-statement: © The Author(s) 2011

==============================

The quality of the *Journal of Orthopaedics and Traumatology* depends on the qualified and regular collaboration of renowned scientists who devoted their time to constructively review the submitted articles. We are indebted to the following experts who reviewed papers which completed the peer-reviewing process within 2011.

Roberto Adani (*Verona*, *Italy*)
Ezio Adriani (*Rome*, *Italy*)
Annunziato Amendola (*Iowa*, *USA*)
Antonio Andreacchio (*Turin*, *Italy*)
Alessandro Aprato (*Turin*, *Italy*)
Paolo Arrigoni (*San Donato Milanese*, *Italy*)
Stefano Artiaco (*Turin*, *Italy*)
Andrea Baldini (*Florence*, *Italy*)
Massimo Balsano (*Vicenza*, *Italy*)
Giovanni Barbanti Brodano (*Bologna*, *Italy*)
Stefano Bastoni (*Milan*, *Italy*)
Vittorio Bellotti (*Barcelona*, *Spain*)
Teresa Benigno (*Turin*, *Italy*)
Celeste Bertone (*San Donato Milanese*, *Italy*)
Giuseppe Bianchi (*Bologna*, *Italy*)
Amin Sadegh Bigham (*Shahrekor*, *Iran*)
Kristian Bjorgul (*Fredrikstad*, *Norway*)
Davide Blonna (*Turin*, *Italy*)
Elena Maria Brach del Prever (*Turin*, *Italy*)
Matteo Cadossi (*Bologna*, *Italy*)
Filippo Calderazzi (*Parma*, *Italy*)
Domenico Campanacci (*Florence*, *Italy*)
Marco Cassini (*Legnago*, *Italy*)
Riccardo Ciatti (*Rome*, *Italy*)
Norberto Confalonieri (*Milan*, *Italy*)
Landino Cugola (*Verona*, *Italy*)
Walter Daghino (*Turin*, *Italy*)
Arpit Dave (*Gurgaon*, *India*)
Orazio De Lucia (*Milan*, *Italy*)
Luigi De Palma (*Ancona*, *Italy*)
Marco De Peppo (*Rome*, *Italy*)
Giacomo Delle Rose (*Rozzano*, *Italy*)
Laura Deriu (*Rome*, *Italy*)
Angelo Dettoni (*Turin*, *Italy*)
Shabir A. Dhar (*Srinagar*, *India*)
Giovanni Luigi Di Gennaro (*Bologna*, *Italy*)
Alberto Di Martino (*Rome, Italy*)
Onofrio Donzelli (*Bologna*, *Italy*)
Pasquale Farsetti (*Rome*, *Italy*)
Flavio Fazioli (*Naples*, *Italy*)
Florian Fischer (*Milan, Italy*)
Olimpio Galasso (*Catanzaro*, *Italy*)
Eduardo García-Rey (*Madrid*, *Spain*)
Alessandro Gasbarrini (*Bologna*, *Italy*)
Giorgio Gasparini (*Catanzaro*, *Italy*)
Federico Grassi (*Novara*, *Italy*)
Tetsuo Hagino (*Kofu Yamanashi*, *Japan*)
Marco Innocenti (*Florence*, *Italy*)
Ernesto Ippolito (*Rome*, *Italy*)
Sujith Konan (*London*, *UK*)
Renato Laforgia (*Bari*, *Italy*)
Danilo Leonetti (*Bologna*, *Italy*)
Giandomenico Logroscino (*Rome*, *Italy*)
Pietro Maniscalco (*Siena*, *Italy*)
Sergio Mapelli (*Milan*, *Italy*)
Ignazio Marcoccio (*Brescia*, *Italy*)
Marcos Almeida Matos (*Salvador de Bahia*, *Brasil*)
Cyril Mauffrey (*Birmingham*, *UK*)
Giovanni Merolla (*Cattolica*, *Italy*)
Jane Christiane Messina (*Milan*, *Italy*)
Maria Teresa Miscione (*Bologna*, *Italy*)
Matteo Nanni (*Bologna*, *Italy*)
Thomasz Nizegorodcew (*Rome*, *Italy*)
Angela Notarnicola (*Bari*, *Italy*)
Masahiko Nozawa (*Tokyo*, *Japan*)
Matthew E. Oetgen (*New Haven*, *CT*, *USA*)
Antonio Pace (*Ancona*, *Italy*)
Paolo Paladini (*Cattolica*, *Italy*)

Matteo Paonessa (*Turin*, *Italy*)
Vinay Parmar (*Manchester*, *UK*)
Vito Pavone (*Catania*, *Italy*)
Alessio Pedrazzini (*Parma*, *Italy*)
Giovanni Peretti (*Milan*, *Italy*)
Giuseppe Peretti (*Milan*, *Italy*)
Franco Pleitavino (*Genoa*, *Italy*)
Bhagwat Singh Rao (*Gujarat*, *India*)
Dario Regis (*Verona*, *Italy*)
Olav Reikerås (*Oslo*, *Norway*)
Giovanni Risitano (*Messina*, *Italy*)
Carlo L. Romanò (*Milan*, *Italy*)
Nicola Santori (*Milan*, *Italy*)
Alfredo Schiavone Panni (*Campobasso*, *Italy*)
Celeste Scotti (*Milan*, *Italy*)
Vincenzo Sessa (*Rome*, *Italy*)
Hamid Reza Shemshaki (*Isfahan*, *Iran*)
Harpreet Singh (*New Delhi*, *India*)
Giuseppe Solarino (*Bari*, *Italy*)
Alasdair G. Sutherland (*Aberdeen*, *UK*)
Pierluigi Tos (*Turin*, *Italy*)
Francesco Traina (*Bologna*, *Italy*)
Federico Trentani (*Bologna*, *Italy*)
Gianluca Vadalà (*Rome*, *Italy*)
Francesca Vannini (*Bologna*, *Italy*)
Manish Kumar Varshney (*New Delhi*, *India*)
Giovanni Vavalle (*Bari*, *Italy*)
Michele Arcangelo Verdano (*Parma*, *Italy*)
Gustavo Zanoli (*Ferrara*, *Italy*)
Giovanni Zatti (*Monza*, *Italy*)

Open Access

This article is distributed under the terms of the Creative Commons Attribution License which permits any use, distribution and reproduction in any medium, provided the original author(s) and source are credited.

